# Supplementary material for: Acute psycho-physiological responses to submaximal constant-load cycling under intermittent hypoxia-hyperoxia vs. hypoxia-normoxia in young males
Source: PeerJ. 2024 Oct 4;12:e18027. doi: 10.7717/peerj.18027 (PMC11457877; doi:10.7717/peerj.18027)
Supplement: Supplemental Information 1 — Values are presented as means ± standard deviations (SD). The coefficient of variance was calculated between-subjects for each hypoxic period and reflects the variability of the SF-index. The SpO2 data were measured after each hypoxic period (i.e., after 4 [P4], 12 [P12], 20 [P20], 28 [P28], and 36 min [P36] of continuous load cycling). IHHT, intermittent hypoxia-hyperoxia; IHT, intermittent hypoxia-normoxia. [file peerj-12-18027-s001.docx]

| **Period** | **IHHT** | | **IHT** | |
| --- | --- | --- | --- | --- |
|  | **Mean ± SD** | **CV** | **Mean ± SD** | **CV** |
| P4 | 573.81 ± 31.37 | 5.47 % | 557.14 ± 54.83 | 9.84 % |
| P12 | 580.00 ± 26.11 | 4.50% | 571.43 ± 27.97 | 4.89 % |
| P20 | 579.05 ± 32.83 | 5.67% | 562.38 ± 27.41 | 4.87 % |
| P28 | 585.71 ± 29.51 | 5.04 % | 580.48 ± 28.27 | 4.87 % |
| P36 | 585.71 ± 29.51 | 3.98 | 580.48 ± 28.27 | 5.21 % |
