# Supplementary material for: Acute psycho-physiological responses to submaximal constant-load cycling under intermittent hypoxia-hyperoxia vs. hypoxia-normoxia in young males
Source: PeerJ. 2024 Oct 4;12:e18027. doi: 10.7717/peerj.18027 (PMC11457877; doi:10.7717/peerj.18027)
Supplement: Supplemental Information 2 — Values are presented as mean differences and 95% confidence intervals and show changes in heart rate at 4 (P4), 8 (P8), 12 (P12), 16 (P16), 20 (P20), 24 (P24), 28 (P28), 32 (P32), 36 (P36), and 40 min (P40) compared to baseline during 40 min of submaximal constant-load cycling under intermittent hypoxia-hyperoxia (IHHT), hypoxia-normoxia (IHT), and sustained normoxia (NOR). [file peerj-12-18027-s002.docx]

| **Period** | **IHHT** | **IHT** | **NOR** |
| --- | --- | --- | --- |
| P4 | 49.4 (41.6 to 57.3),  p < 0.001, d = 3.75 | 50.1 (42.2 to 57.9),  p < 0.001, d = 3.80 | 43.4 (35.5 to 51.2),  p < 0.001, d = 3.30 |
| P8 | 44.6 (36.7 to 52.5),  p < 0.001, d = 3.38 | 50.8 (42.9 to 58.7),  p < 0.001, d = 3.85 | 49.3 (41.4 to 57.2),  p < 0.001, d = 3.74 |
| P12 | 58.0 (50.2 to 65.9),  p < 0.001, d = 4.40 | 59.9 (52.0 to 67.7),  p < 0.001, d = 4.54 | 52.6 (44.7 to 60.4),  p < 0.001, d = 4.00 |
| P16 | 50.1 (57.9 to 42.2),  p < 0.001, d = 3.80 | 56.7 (48.8 to 64.6),  p < 0.001, d = 4.30 | 54.5 (46.6 to 62.4),  p < 0.001, d = 4.13 |
| P20 | 62.5 (54.6 to 70.4),  p < 0.001, d = 4.74 | 64.4 (56.6 to 72.3),  p < 0.001, d = 4.89 | 57.3 (49.4 to 65.2),  p < 0.001, d = 4.35 |
| P24 | 54.0 (46.1 to 61.9),  p < 0.001, d = 4.10 | 60.9 (53.0 to 68.7),  p < 0.001, d = 4.62 | 57.9 (50.0 to 65.8),  p < 0.001, d = 4.40 |
| P28 | 64.8 (56.9 to 72.6),  p < 0.001, d = 4.91 | 67.3 (59.4 to 75.2),  p < 0.001, d = 5.11 | 59.8 (51.9 to 67.6),  p < 0.001, d = 4.54 |
| P32 | 56.5 (48.6 to 64.4),  p < 0.001, d = 4.29 | 63.4 (55.5 to 71.2),  p < 0.001, d = 4.81 | 61.1 (53.2 to 69.0),  p < 0.001, d = 4.63 |
| P36 | 67.8 (59.9 to 75.6),  p < 0.001, d = 5.14 | 69.8 (61.9 to 77.7),  p < 0.001, d = 5.30 | 62.9 (55.0 to 70.7),  p < 0.001, d = 4.77 |
| P40 | 58.4 (50.6 to 66.3),  p < 0.001, d = 4.43 | 64.9 (57.0 to 72.8),  p < 0.001, d = 4.92 | 62.6 (54.8 to 70.5),  p < 0.001, d = 4.75 |
